# Clinical Significance of Screening Differential Metabolites in Ovarian Cancer Tissue and Ascites by LC/MS

**DOI:** 10.3389/fphar.2021.701487

**Published:** 2021-11-01

**Authors:** Miao Liu, Yu Liu, Hua Feng, Yixin Jing, Shuang Zhao, Shujia Yang, Nan Zhang, Shi Jin, Yafei Li, Mingjiao Weng, Xinzhu Xue, Fuya Wang, Yongheng Yang, Xiaoming Jin, Dan Kong

**Affiliations:** ^1^ Department of Pathology, Harbin Medical University, Harbin, China; ^2^ Department of Pathology, Beidahuang Industry Group General Hospital, Harbin, China; ^3^ Department of Gynecology, Tumor Hospital of Harbin Medical University, Harbin, China

**Keywords:** ovarian cancer and ascites, metabonomics, differential metabolites, lipid metabolism, tumor markers

## Abstract

Tumor cells not only show a vigorous metabolic state, but also reflect the disease progression and prognosis from their metabolites. To judge the progress and prognosis of ovarian cancer is generally based on the formation of ascites, or whether there is ascites recurrence during chemotherapy after ovarian cancer surgery. To explore the relationship between the production of ascites and ovarian cancer tissue, metabolomics was used to screen differential metabolites in this study. The significant markers leading to ascites formation and chemoresistance were screened by analyzing their correlation with the formation of ascites in ovarian cancer and the clinical indicators of patients, and then provided a theoretical basis. The results revealed that nine differential metabolites were screened out from 37 ovarian cancer tissues and their ascites, among which seven differential metabolites were screened from 22 self-paired samples. Sebacic acid and 20-COOH-leukotriene E4 were negatively correlated with the high expression of serum CA125. Carnosine was positively correlated with the high expression of serum uric acid. Hexadecanoic acid was negatively correlated with the high expression of serum γ-GGT and HBDH. 20a,22b-Dihydroxycholesterol was positively correlated with serum alkaline phosphatase and γ-GGT. In the chemotherapy-sensitive and chemotherapy-resistant ovarian cancer tissues, the differential metabolite dihydrothymine was significantly reduced in the chemotherapy-resistant group. In the ascites supernatant of the drug-resistant group, the differential metabolites, 1,25-dihydroxyvitamins D3-26, 23-lactonel and hexadecanoic acid were also significantly reduced. The results indicated that the nine differential metabolites could reflect the prognosis and the extent of liver and kidney damage in patients with ovarian cancer. Three differential metabolites with low expression in the drug-resistant group were proposed as new markers of chemotherapy efficacy in ovarian cancer patients with ascites.

## Introduction

Cancer is a metabolic disease characterized by metabolic reprogramming of cells, which is essential to maintain a high proliferation rates and resist changes in cell death signals (Wishart, 2019; Wang et al., 2020). The characteristics of abnormal tumor metabolism may become a new indicator to monitor the condition of tumor patients, and thus contribute to the early diagnosis of tumor. In addition, monitoring the metabolic status of patients is helpful to understand the mechanism of action of some anticancer therapies, find new targets for tumor therapy and evaluate the prognosis of diseases (Le et al., 2019). Metabonomics is a rapid and efficient approach to identify novel cancer biomarkers, which has emerged as a complementary technology of genomics and proteomics. It integrates high-throughput and high-resolution analytical techniques with bioinformatics to study the metabolic level of organisms and provides a unique perspective for understanding biology, and its applications in oncology research promotes the discovery of biomarkers in disease and medical therapy target (Antony et al., 2019; Chen et al, 2019a). Ultra performance liquid chromatography time of flight mass spectrometry (UPLC-Q-TOF MS) is an effective method developed in recent years for the analysis of complex samples, especially suitable for the separation of trace complex mixtures and high throughput studies (Xu et al., 2019; Zhang et al., 2019).

In metabolomics studies of ovarian cancer, serum or plasma, urine and cell samples are often used to screen potential biomarkers (Lu et al., 2019; Yang et al., 2019). In recent years, ascites have been gradually applied to metabolomics detection due to the characteristics of metabolic changes and heterogeneity of ascites microenvironment. Ovarian cancer with ascites formation is a common complication in the middle and late stage, for which chemotherapy is the main means of treatment (Han et al., 2019). However, some ovarian cancer patients still develop ascites repeatedly after chemotherapy, suggesting the emergence of chemotherapy resistance. Whether differential metabolites from ascites of ovarian cancer could be screen out by means of metabolomics, which are of significance to chemotherapy sensitivity or drug resistance, and then used to guide clinical diagnosis and treatment?

The design of this study was to test the selected ovarian cancer ascites samples together with their corresponding surgically resected ovarian cancer tissues. From multiple single-case analyses, common differential metabolites were identified in ovarian cancer ascites and tissues. And different differential metabolites were screened from ovarian cancer ascites samples between chemotherapy group and non-chemotherapy group, chemotherapy-sensitive group and chemotherapy-resistant group. In particular, formalin-fixed paraffin-embedded (FFPE) ovarian cancer tissue should be used for metabolomics testing because recurrent ascites are usually produced after surgical resection of the cancer tissue. FFPE tissue is not a routine metabolomics sample, but it has been reported that significant differential metabolites have been obtained from them (Arima et al., 2010; Feng et al., 2019).

In this study, UPLC-Q-TOF MS technology was used to screen and analyze differential metabolites between the two groups vertically and horizontally, especially to find the differential metabolites related to chemotherapy sensitivity and chemotherapy resistance, expecting to provide new clues and new hope in guiding clinical diagnosis and treatment.

## Materials and Methods

### Samples of ovarian Cancer Tissue and Ascites Collection

A total of 82 cases of high-grade serous ovarian cancer with ascites were collected in the Department of Gynecology of the Affiliated Cancer Hospital of Harbin Medical University from November 2014 to June 2019 (Supplementary Table S1). The ascites samples from 37 ovarian cancer patients in recent three years were selected and divided into non-chemotherapy group (N) and platinum chemotherapy group (C). The patients who underwent chemotherapy were further divided into the chemotherapy-sensitive group (≥6 months for platinum sensitivity, CS) and the chemotherapy-resistant group (<6 months is platinum resistance, CR) (Cortez et al., 2018). Of the 37 patients with ovarian cancer and ascites, 22 cases had the corresponding ovarian cancer tissue and ascites supernatant samples, 12 cases had the corresponding ovarian cancer tissue, ascites supernatant and precipitate cell samples. All cases had complete clinicopathological data and written informed consent. This study was approved by the ethics committee of The Third Affiliated Hospital of Harbin Medical University.

### Samples of ovarian Cancer Tissue and Ascites Preparation

The supernatant of ascites from ovarian cancer was collected into 1.5 ml centrifuge tube. 100 μL of sample were mixed with 4 times the volume of methanol (cooled to −80 °C). The mixture was vortexed for 30 s and allowed to stand for 1 h at −20°C and was centrifuged at 14,000 × *g* for 10 min. For Precipitated cell samples, moderate ACK lysis (Leagene Biotechnology, China) buffer was added to the ascites precipitated cells and were lysed for 10 min, centrifuged at 3,500 r/min for 5 min, and then collected into a 1.5 ml centrifuge tube. 10.00 ± 1.00 mg of sample were homogenized with ceramic beads in 1.5 ml methanol for two times using a homogenizer. The homogenization took 20 s with 5 s intervals each time, and ice was used to keep the low temperature of homogenization. The mixture was centrifuged at 14,000 × *g* for 10 min. For formalin-fixed tissue samples, 20 μm FFPE sections are extracted from the tissue block about 30–40 μm, mixed with 1.5 ml of 80% methanol, and incubated at 70 °C for 30–45 min without any deparaffinization procedure. Then the mixture allowed to stand for 15 min on ice and was centrifuged at 14,000 × *g* for 15 min (Yuan et al., 2012). The supernatants of both ascites and FFPE samples after centrifugation were leached using a 0.22 µm filter membrane and centrifuged at 14,000 × *g* for 10 min again. The supernatant was dried with nitrogen. The quality control (QC) sample was a pooled sample prepared by mixing aliquots of all samples across different groups. All samples reconstituted with 100 μl of 75% acetonitrile.

### LC-MS Data Acquisition

The LC–MS data was fulfilled by an ultrahigh performance liquid chromatography (UPLC) system (1,200 infinity series, Agilent Technologies, United States) coupled to quadruple time-of-flight (Q-TOF) mass spectrometer (Agilent 6,530, Agilent Technologies, United States) in positive and negative ion modes. LC separation is achieved in Agilent SB-C18 column (particle size, 1.8 µm; 100 mm (length) × 2.1 mm (i.d.)) held at 40°C. The chromatographic conditions were as follows: flow rate of 0.3 ml/min; sample injection volume, 10 µl. ESI+: mobile phases A, 0.1% FA in ACN; and mobile phase B, 0.1% FA in water; ESI-: mobile phases A, ACN; and mobile phase B, water. Linear elution gradient program was set as follows: 0–1 min: 5% B; 1–10 min: 5% B to 95% B; 10–13 min: 95% B; 13–13.1 min: 95% B to 5% B; 13.1–20 min: 5% B. The acquisition rate of MS data acquisition was set at two spectra/s and the TOF mass range was set at m/z 50–1000Da. Other parameters were set as follows: dry gas temperature: 350°C; dry gas flow:10 L/min; nebulizer pressure: 50psi; Oct RFV, 750 V; and fragmentor voltage: 120 V. capillary voltage: 4000 V and −3500 V for positive and negative, respectively. QC samples were analyzed every five injections of biological samples and a blank sample (ACN: H2O, 1:1, v/v) to monitor the stability of the data acquisition and used for data normalization.

### Data Processing

Raw MS data (.d) files are preprocessed by XCMS online (Forsberg et al., 2018). Further data pre-processing including missing value estimation, Log transformation were performed by MetaboAnalyst 4.0 (Chong et al., 2018). The generated data matrix consisted of the mass-to-charge ratio (m/z) value, retention time (RT), and peak abundance. Multivariate statistical analysis (PCA and PLS-DA) was carried out by SIMCA-P software package (14.0). The permutation test of the model is carried to avoid the overfitting. The variable important in the projection (VIP) value was calculated for each variable in the PLS-DA model. In addition, an independent *t*-test was used for comparison. The differential metabolites with *p* value less than 0.05 and VIP value greater than 1 were selected.

Spearman test was used to analyze the correlation between differential metabolites and clinicopathological data of 82 patients with ovarian cancer and ascites, and 22 self-matched samples were analyzed between groups.

The metabolites were preliminarily annotated by matching the MS/MS spectrum and precise mass of metabolites with the structural information of metabolites in Human Metabolite database (HMDB). Pathway enrichment was performed using MetaboAnalyst 4.0 and the KEGG database. Kaplan-Meier survival analysis was used to evaluate the relationship between differential metabolite-associated enzyme genes and prognosis in patients with ovarian cancer. Random forest analysis was set up in MetaboAnalyst 4.0 to estimate the significance of each differential metabolite.

### Statistical Analyses

Clinical data are shown as the mean ± SD. Statistical analyses were performed using SPSS 21.0 software. Comparisons between two or multiple parameters were performed using the chi-square test. Statistical significance was considered to exist at *p* < 0.05. The receiver operating characteristic (ROC) was used to evaluate the diagnostic performance of metabolite biomarkers. The larger the AUC of the area under the curve is, the higher the diagnostic value is, and the AUC is usually between 0.5 and 1.

## Results

### Metabolic Profiles of ovarian Cancer Tissue and Ascites

**FIGURE 1:**
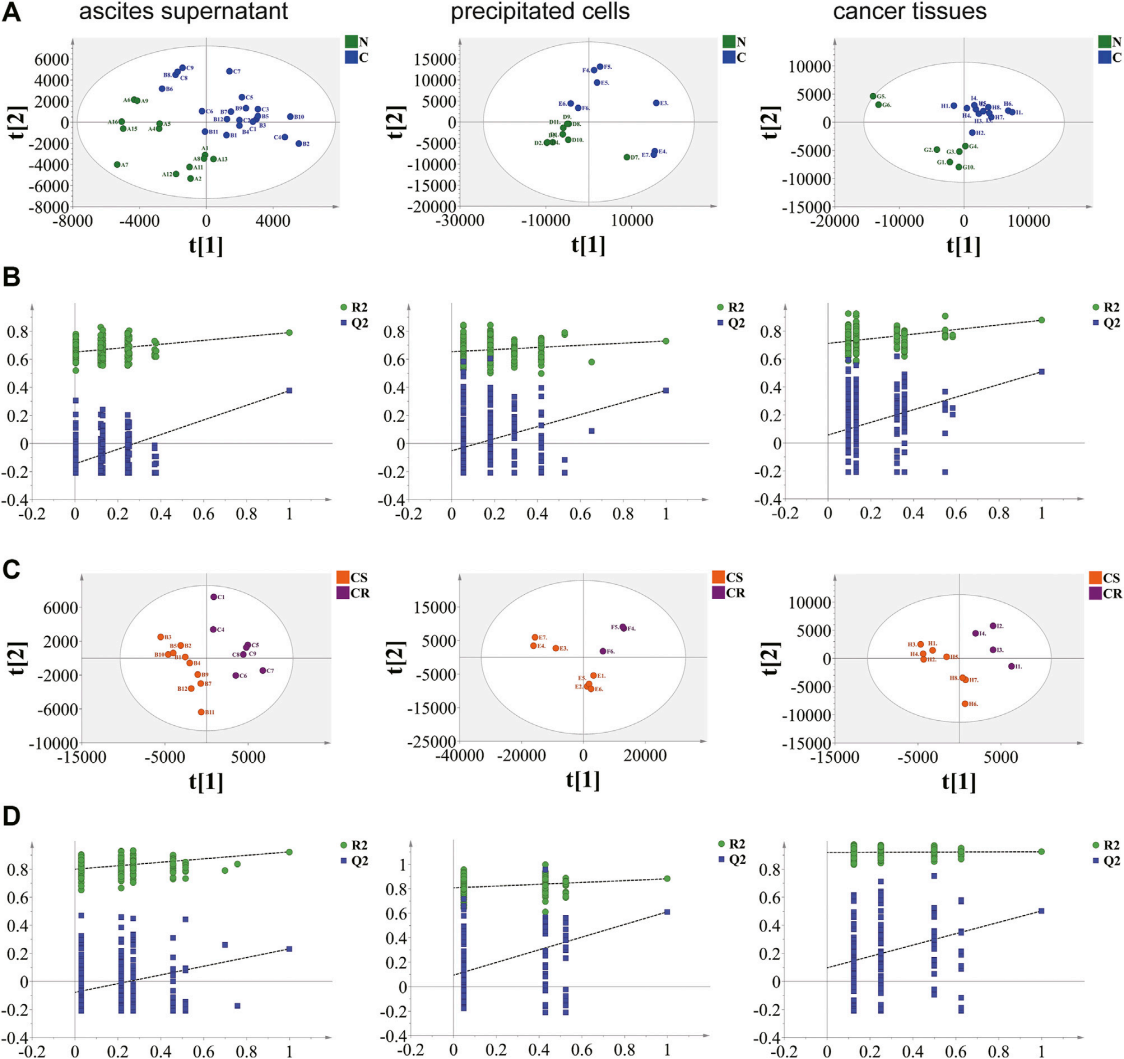
Results of supervised partial least squares discriminant analysis (PLS-DA) and permutation test. **(A)**, **(B)** non-chemotherapy group and chemotherapy group. **(C)**, **(D)** chemotherapy-sensitivity group and chemotherapy-resistant group. Ovarian cancer ascites supernatant, precipitated cells and tissue samples can be separated. The permutation test indicates that the PLS-DA model of tissue data set is not over-fitting.

A total of 11,502, 7,591 and 10,053 metabolic ion peaks were detected in ovarian cancer tissue, ascites supernatant and precipitated cells, respectively. To select the differential metabolites between non-chemotherapy group vs chemotherapy group and Chemotherapy-sensitive group and chemotherapy-resistant group, unsupervised principal component analysis (PCA) was first applied to visualize the metabolomics data. Apparent differences between the metabolic profiles of ovarian cancer tissue, ascites supernatant and precipitated cell samples was observed among each group from the PCA score plot. Tight clustering of QC samples demonstrated that the data quality is repeatability and stability (Supplementary Figure S1). The supervised PLS-DA model showed that the samples achieved significant separation between the non-chemotherapy group vs chemotherapy group and Chemotherapy-sensitive group and chemotherapy-resistant group, indicating that different kinds of samples had different metabolic characteristic peaks among groups, and permutation tests showed that the supervised models were not overfitted (Figure 1).

### Select Differential Metabolites in ovarian Cancer Tissues and Ascites

**FIGURE 2:**
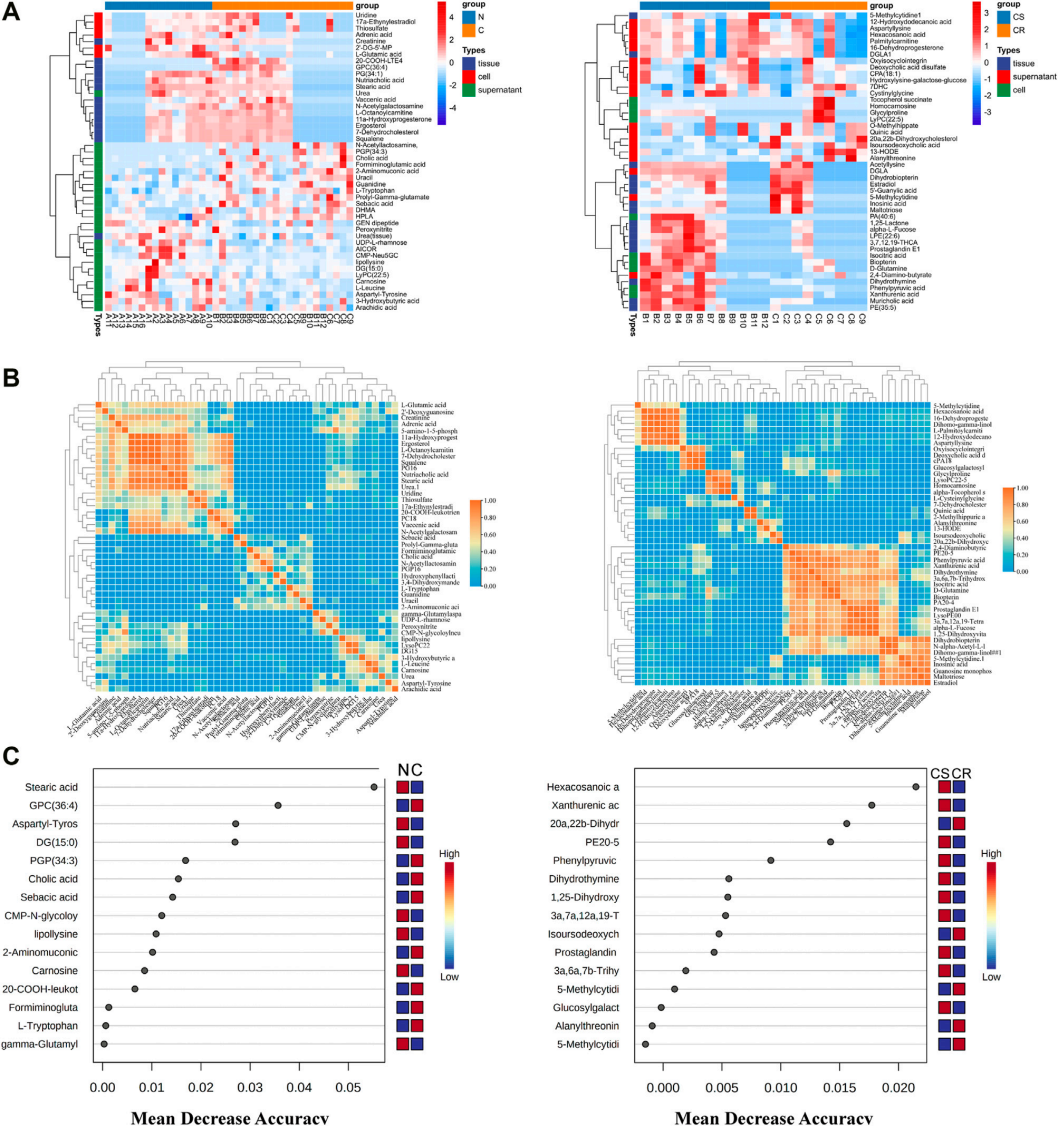
Metabolites Characteristics of ascites supernatant, precipitated cells and tissues in ovarian cancer. **(A)** Results of hierarchical cluster analysis of metabolites of ascites supernatant, precipitated cells and cancer tissue in ovarian cancer. Each row represents a metabolite, and each column represents a different group. **(B)** Correlation heatmap of differential metabolites in N vs C **(left)** and CS vs CR **(right)**. **(C)** Random forests show important differential metabolites in N vs C **(left)** and CS and CR **(right)**. N: non-chemotherapy group, C: chemotherapy group; CS: chemotherapy-sensitive group; CR: chemotherapy-resistant group.

Differential metabolites were obtained between the non-chemotherapy group and chemotherapy group, chemotherapy-sensitive group and chemotherapy-resistant group of the three kinds of samples (Supplementary Table S2). Heatmap of the relative intensity with differential metabolites showed that they were well separation between groups (Figure 2A). Correlation analysis showed that there was a certain correlation between them (Figure 2B). These differential metabolites were also screened in samples of ovarian cancer tissue and its own paired ascites. At the same time, shared differential metabolites between the ascites and cancer tissue were screened in the paired samples. The differential metabolites with higher significance were selected by random forest analysis between non-chemotherapy and chemotherapy, and between chemotherapy-sensitive and chemotherapy-resistant groups (Figure 2C). Similarly, the shared differential metabolites in self-matched samples were intersected with random forest results to further screen the differential metabolites.

**FIGURE 3:**
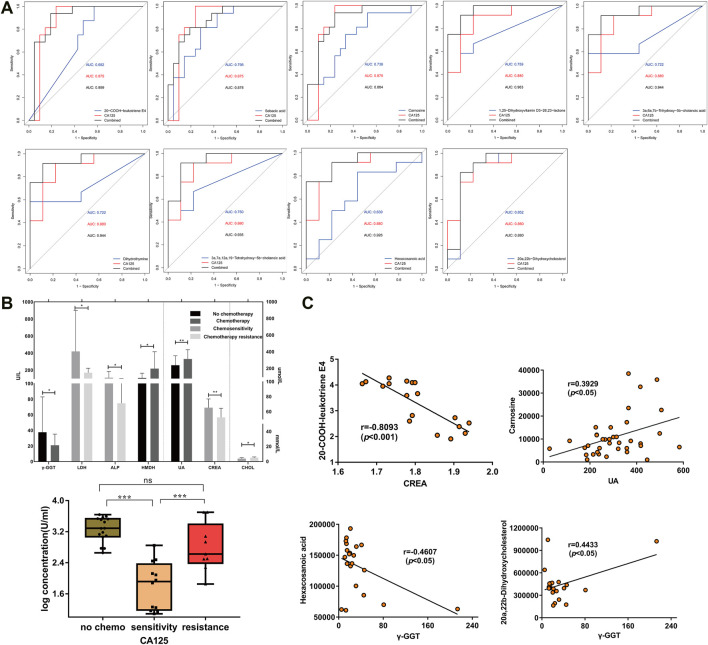
Analysis of differential metabolites and clinical data of patients. **(A)** ROC curves of nine metabolites combined with CA125 respectively. **(B)** The expression of serological markes in non-chemotherapy group vs chemotherapy group and chemotherapy-sensitivity group vs chemotherapy-resistant group (*p* < 0.05). The ordinate is the concentration (log). γ-GGT: γ-Gltamyltranspeptidase; LDH: Lactic dehydrogenase; ALP: Alkaline phosphatase; HBDH: Hydroxybutyrate dehydrogenase; UA: Uric Acid; CREA: Creatinine; CHOL: Cholesterol. **(C)** Correlation analysis scatter plot for 20-COOH-LTE4 and creatinine, carnosine and uric acid, 20a, 22b-dihydroxycholesterol, hexadecanoic acid and γ-GGT (*p* < 0.05).

ROC analysis showed that the distinguishing ability of metabolites was less than that of CA125, but when combined with CA125, nine metabolites increased. They are carnosine, 20-COOH-leukotriene E4, sebacic acid in non-chemotherapy group vs chemotherapy group; 1,25-dihydroxyvitamin D3-26,23-lactone, 20a,22b-dihydroxycholesterol, 3a,6a,7b-trihydroxy-5b-cholicacid, 3a,7a,12a,19-tetrahydroxy-5b-cholic acid, dihydrothymine, hexadecanoic acid in Chemotherapy-sensitive group and chemotherapy-resistant group (Figure 3A). The combination of metabolites and CA125 may improve the predictive value of ovarian cancer diagnosis. In the paired samples, a total of 7 differential metabolites had a good diagnostic value and they were included in nine differential metabolites.

### Correlation Between nine Differential Metabolites and Clinical Pathological Data

The serum biochemical indexes of 82 patients with ovarian cancer showed that serum CA125, uric acid and α-hydroxybutyrate dehydrogenase (HBDH) were significantly decreased, γ-glutamyl transpeptidase (γ-GGT) was significantly increased in chemotherapy group, serum CA125, creatinine, lactate dehydrogenase (LDH), alkaline phosphatase (ALP) were increased, and serum cholesterol was significantly decreased in chemotherapy-resistant group (*p* < 0.05, Figure 3B). Further analysis of the correlation between metabolites and serological indexes showed that 20-COOH-leukotriene E4 and sebacic acid were negatively correlated with serum CA125 (*p* < 0.05), 20-COOH-leukotriene E4 was negatively correlated with serum creatinine (*p* < 0.05), carnosine was positively correlated with serum uric acid (*p* < 0.05), 20a,22b-Dihydroxycholesterol was positively correlated with serum ALP and γ-GGT (*p* < 0.05), and hexadecanoic acid was negatively correlated with serumγ-GGT and HBDH (*p* < 0.05), and other metabolites were not significantly correlated with serum biochemical indexes (Figure 3C and Supplementary Figure S3).

### Survival Analysis of Genes Related to Differential Metabolites

**FIGURE 4:**
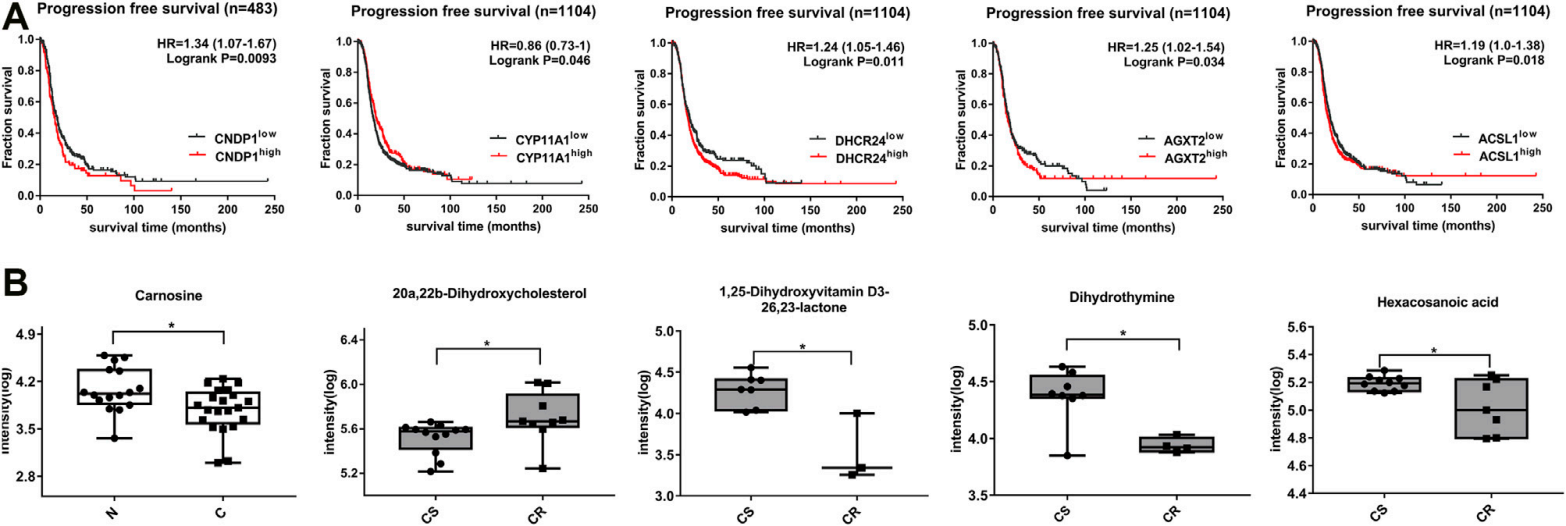
Expression of differential metabolites and correlation between related enzyme genes and progression-free survival of patients. **(A)** Progression-free survival curve of differential metabolites (*p*< 0.05). **(B)** Box plots of corresponding metabolite biomarkers in non-chemotherapy group vs chemotherapy group (blank) and chemotherapy-sensitive group vs chemotherapy-resistant group (gray) (*p*< 0.05).

The related enzymes genes of differential metabolites were obtained by KEGG database (Table 1). In addition, 1,232 patients with high-grade serous ovarian cancer from Kaplan-Meier database were selected to analyze the relationship between differential metabolite-related enzyme gene expression level and prognosis of ovarian cancer patients. Progression free survival analysis (PFS) showed that the high expression of enzyme gene had a poor prognosis, which were CNDP1 (carnosine), LTC4S (20-COOH-LTE4), CYP11A1 (20a, 22b-dihydroxy cholesterol), CYP7A1 (W-Muricholic acid), CYP27A1 (3,7,12,19-THCA) (*p* < 0.05). Low expression has a poor prognosis, which are ACADL (sebacic acid), DHCR24 (1,25-lactone), AGXT2 (dihydrothymine), ACSL1 (hexadecanoic acid) (*p* < 0.05, Figure 4A, Supplementary Figure S2A). Carnosine that is increased in non-chemotherapy group is also higher in patients with poor disease-free survival. Similarly, among drug-resistant patients, increased 20a,22b-dihydroxycholesterol is also higher in patients with poor progression-free survival, and decreased 1,25-lactone, dihydrothymine and hexadecanoic acid are also lower in patients with poor progression-free survival (Figure 4B, Supplementary Figure S2B). In addition, LTC4S, CYP11A1, CYP27A1, ACSL1, and AGXT2 all showed significance in overall survival and progression-free survival analysis (*p* < 0.05).

**TABLE 1:** Biological function of potential biomarkers and related enzyme genes in ovarian cancer.

Metabolites	Log FC	CA125 + AUC	Gene	KM *p*-value	Poor survival	Pathway	Function
Carnosine	0.65	0.884	CNDP1	< 0.001	High	Histidine metabolism	Apoptosis
20-COOH-LTE4	−0.28	0.899	LTC4S	0.012	High	Arachidonic acid metabolism	Angiogenesis
Sebacic acid	0.2	0.879	ACADL	< 0.001	Low	Fatty acid metabolism	Inhibit metastasis
Calcitriol lactone	−0.66	0.963	DHCR24	0.011	Low	Steroid biosynthesis	Inhibit proliferation
20a,22b-Dihydroxycholesterol	0.3	0.88	CYP11A1	0.046	High	Steroid hormone biosynthesis	Proliferation
Muricholic acid	−0.28	0.944	CYP7A1	0.032	High	bile acid biosynthesis	Proliferation drug resistance
3,7,12,19-THCA	−0.47	0.935	CYP27A1	0.01	high	bile acid biosynthesis	Proliferation drug resistance
Dihydrothymine	−0.48	0.88	AGXT2	0.034	low	Pyrimidine metabolism	Anti-apoptosis drug resistance
Hexacosanoic acid	−0.14	0.926	ACSL1	0.018	low	Fatty acid metabolism	Proliferation drug

### Pathway Analysis of Differential Metabolites in ovarian Cancer Tissue and Ascites

**FIGURE 5:**
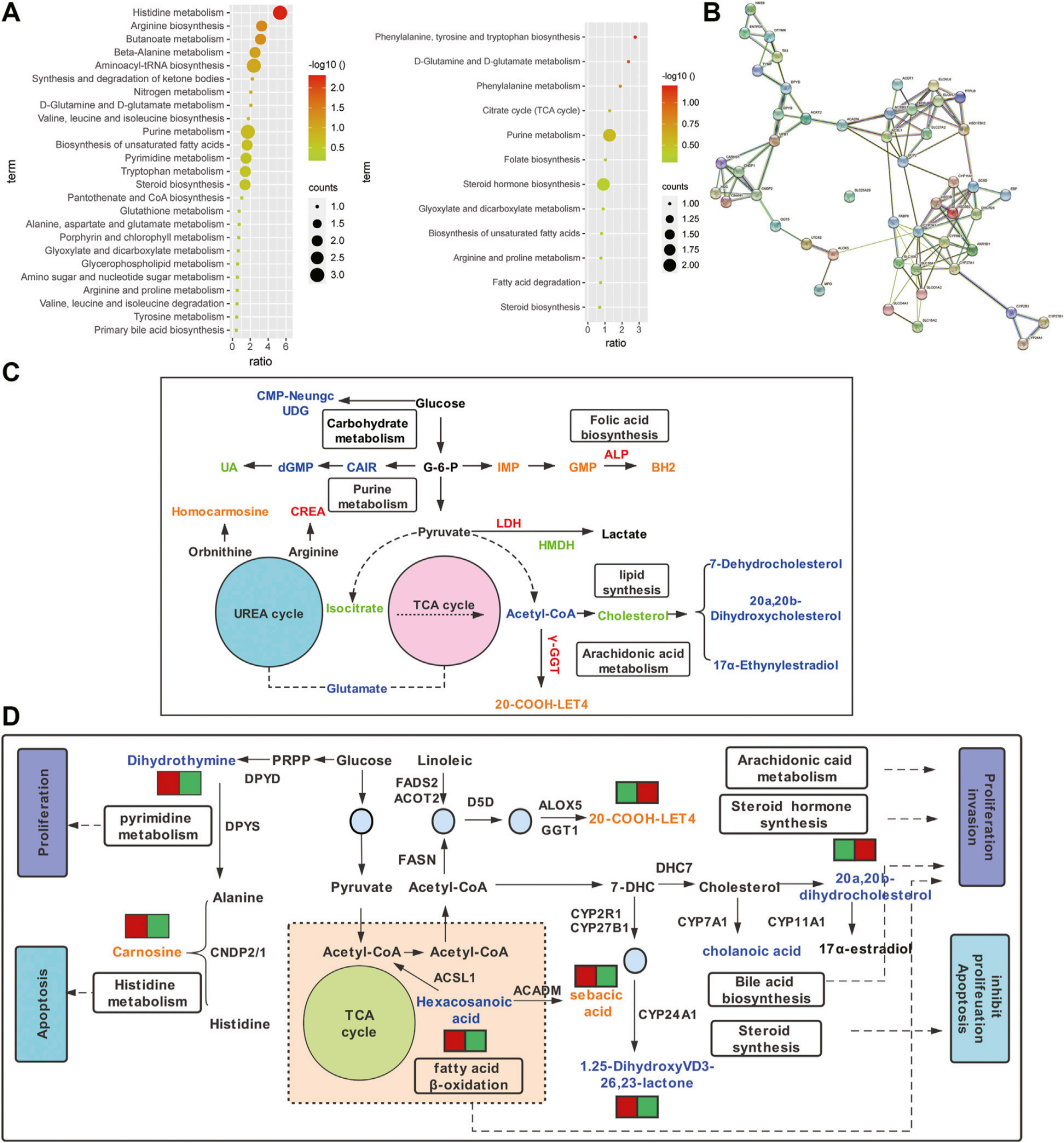
Pathway analysis of differential metabolites between ovarian cancer tissue and ascites. **(A)** Enrichment pathway bar chart of differential metabolites in non-chemotherapy group vs chemotherapy group **(left)** and chemotherapy-sensitive group and chemotherapy-resistant group **(right)**. **(B)** The shared pathways involved in differential metabolites and serological indicators. Orange or blue represent up-regulated or down-regulated differential metabolites, respectively. Red or green represent up-regulated or down-regulated serological indexes, respectively. **(C)** Network view of STRING analysis of genes related to differential metabolites (*p*< 0.001). **(D)** Proposed metabolic mechanisms associated with ovarian cancer. Red font represents the non-chemotherapy group and chemotherapy group, which is mainly involved in histidine metabolism and fatty acid β oxidation, promoting apoptosis and inhibiting metastasis. Blue font represents the chemotherapy-sensitivity group and chemotherapy-resistant group, which are mainly involved in lipid metabolism, promoting tumor proliferation and metastasis, and the occurrence of drug resistance. 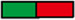
denote down-regulation/up-regulation.

Metabolite pathway analysis showed that 25 and 12 pathways were enriched in non-chemotherapy group vs chemotherapy group, chemotherapy-sensitive group vs chemotherapy-resistant group (Figure 5A, Supplementary Table S3). There were 6 common pathways, which were biosynthesis of unsaturated fatty acids, steroid biosynthesis, d-Glutamine and d-glutamate metabolism, glyoxylate and dicarboxylate metabolism, purine metabolis and arginine and proline metabolism. Differential metabolites and clinical serological indicators also participate in the same pathway, including arachidonic acid, purine, arginine, folate metabolism and steroid synthesis, et al. (Figure 5B). The change trend of metabolic pathways is consistent. This may explain that changes in metabolites in cancer tissue not only lead to changes in ascites metabolites, but also cause serological changes.

### The Potential Mechanism of Differential Metabolites in the Progression of ovarian Cancer

The STRING protein interaction network analysis of the differential metabolite-associated enzyme genes was performed, which had significant correlation (*p* < 0.001, Figure 5C). KEGG enrichment analysis showed that it was mainly involved in the PPAR signaling pathway, steroid biosynthesis, fatty acid metabolism, secondary bile acid biosynthesis, et al. Molecular pathways that may be involved in ovarian cancer were constructed (Figure 5D). Metabolites and related enzymes may play a role in the progression of ovarian cancer and the development of drug resistance, including malignant proliferation, invasion, apoptosis, et al. (Table 1).

## Discussion

Metabonomics, as an emerging histological technology, can elucidate the occurrence and development of some diseases by exploring the relationship between changes in metabolite content and biological phenotypes and provide important information for the discovery of therapeutic targets (Kaushik and DeBerardinis, 2018). In recent years, it has been widely applied in screening tumor prognostic markers and is also a prospective research method (Arnaud et al., 2020; Loftfield et al., 2020; Wu et al., 2020).

Biological samples, including urine, plasma or serum, saliva, extracts of cells and tissues, tissue (usually fresh tissue), have been applied to metabolism-based cancer research (Yang et al., 2018a; Plewa et al., 2019; Govorov et al., 2020). Compared with urine and plasma, ascites produced from ovarian cancer is more reflective of the occurrence, development and prognosis of ovarian cancer (Bachmayr-Heyda et al., 2017 **;** Zennaro and Nicolè, 2020). In this study, differential metabolites were obtained by metabonomics detection on 37 ovarian cancer ascites samples and ovarian cancer tissue samples paired with ascites. Nine differential metabolites selected were mainly involved in fatty acid metabolism. That finding was basically consistent with the results of Anna et al. in the ascites metabolism of ovarian cancer patients that the decrease of lipids and essential amino acids was directly related to tumor metabolism and can promote cell proliferation and growth (Bachmayr-Heyda et al., 2017). Ovarian cancer ascites constituted a unique tumor microenvironment. The high free fatty acid content in ascites provided a huge energy source and may force ovarian cancer cells to undergo metabolic reprogramming from aerobic glycolysis to fatty acid β-oxidation to produce energy for tumor growth **(** Gong et al., 2020; Yang et al, 2018b; Chen et al., 2019b). Chemotherapy can induce hypoxia in tumor tissues, increase the absorption of free fatty acids, and provide energy for the proliferation of cancer cells (Stoykova and Schlaepfer, 2019; Iwamoto et al., 2018). This study also found that 20-COOH-leukotriene E4 and sebacic acid were upregulated in patients after chemotherapy. 20-COOH-leukotriene E4 belongs to arachidonic acid, which can inhibit cancer cell growth by activating peroxisome proliferator receptor (PRPP) and inducing the production of prostaglandin D2 (PGD2) (Alves et al., 2019). In this study, 20-COOH-leukotriene E4 was up-regulated in cancer tissues after chemotherapy, which may be associated with enhanced γ-GGT activity after chemotherapy, leading to increased synthesis, consistent with elevated serum γ-GGT in post-chemotherapy patients. Sebacic acid is a medium chain acyl-coenzyme A dehydrogenase (ACADM), metabolite that is present in a variety of tumors, such as squamous cell carcinoma of the skin and melanoma (Fukumoto et al., 2017; Li et al., 2019). Lack of ACADM indirectly leads to the increase of sebacic acid in patients after chemotherapy.

Previous studies have shown that EOC with ascites formation or recurrence is caused by chemotherapy resistance (Ahmed et al., 2016). Some scholars found that the serum levels of lipid metabolites (lysophosphatidylcholine, lysophosphatidylethanolamine) and fatty acids (hydroxyphenyllactic acid, 2-octenoic acid) were significantly increased in patients with recurrent ovarian cancer through the study of plasma metabolomics (Ke et al., 2016). Hexadecanoic acid was a product of fatty acid elongation. Fatty acid prolonging enzyme over expression in cancer promoted peroxisome β oxidation, relatively increased fatty acid uptake and utilization, and promoted drug resistance (Mika et al., 2017; Prusinkiewicz et al., 2020). This study found that hexadecanoic acid was down-regulated in the drug-resistant group of ovarian cancer, indicating that the generation of chemotresistance can promote the uptake and utilization of hexadecanoic acid, leading to the enhancement of its lipid catabolites. Previous studies have reported that dysregulation of cholesterol homeostasis in cancer can up-regulate the transcription of genes related to glycolysis and lipid synthesis, and further promote tumor proliferation, invasion and metastasis (Ahmad et al., 2019; Nazih and Bard, 2020). This was consistent with the results of this study. In the ascites of the chemotherapy-resistant group, Muricholic acid, 3,7,12,19-THCA, 20a, 22b-dihydroxy cholesterol, 1,25-dihydroxyvitamin D3-26, 23-lactone were significantly dysregulated. These results suggested that cholesterol, as a precursor of bile acid and steroid hormones, can promote cancer cell proliferation, invasion and inhibit apoptosis (Ahmad et al., 2019). The increasing evidence also suggests that carcinogenesis was associated with metabolic reprogramming of the pyrimidine degradation pathway. Dihydrothymine, a decomposition product of thymine, was also considered as a potential cytotoxic metabolite and was up-regulated in both early lung adenocarcinoma and breast cancer (Basbous et al., 2020; Wikoff et al., 2015; Shaul et al., 2014), but in this study, it was found to be downregulated in the chemotherapy-resistant group, which may be associated with enhanced activity of the degradation enzyme dihydropyrimidase (DHP).

The clinical data analysis of 82 cases of ovarian cancer patients showed that the incidence of diabetes was higher in the group of ovarian cancer ascites resistance. Studies have shown that hyperglycemia can induce oxidative stress and DNA damage and promote tumor genesis and development (Suh and Kim, 2019). In the process of peritoneal metastasis, glycolytic enzymes such as LDH and glucose transporter (GLUT) were activated to promote glycolysis and provide energy and material requirements for cancer cell proliferation (Archid et al., 2019). In this study, serum LDH, ALP and γ-GGT were up-regulated in chemotherapy-resistant group, and serum HBDH was up-regulated in non-chemotherapy group. These metabolic enzymes that promote malignant transformation were expected to be therapeutic targets. In addition, the correlation analysis of differential metabolites with serum CA125 and clinical serological indicators indicated that differential metabolites could indirectly reflect the liver and kidney metabolism and chemotherapy efficacy of ovarian cancer patients. Enrichment analysis of KEGG pathway revealed that significant dysregulation of metabolic pathways such as nucleotide metabolism, fatty acid metabolism and PPAR signaling pathway. These results indicated that metabolic reprogramming occurred in ovarian cancer patients after chemotherapy and drug resistance, which lead to the alterations in hepatic and renal metabolism. It was suggested that the detection of the expression of these different metabolites was helpful to the timely revision of clinical treatment plan.

In addition, differential metabolites screened from paired ovarian cancer samples were included in unpaired ovarian cancer ascites samples, especially hexadecanoic acid, 1, 25-dihydroxyvitamin D3-26, 23-lactone and dihydrothymine were significantly down-regulated in the chemotherapy-resistant group. These results suggested that they could be used as predictors of whether ovarian cancer patients product ascites after surgery and were resistant to current first-line chemotherapy drugs. To date, these differential metabolites screened in ascites or tissues are proposed for the first time in ovarian cancer. Their involvement in the progression of ovarian cancer and the mechanism of ascites chemotherapeutic resistance remain to be further explored in depth.

To further investigate the role of these differential metabolites in the development of ovarian cancer, this study constructed the potential molecular function network of differential metabolites and found that these differential metabolites play different roles in the metabolic disorders of ovarian cancer. Ovary cancer cells use lipid metabolism in ascites or retinal microenvironment to enhanced ovarian cancer metastasis and aggressiveness through different signaling pathways, such as lipogenesis and signaling activation mediated by AMPK, enhanced arachidonic acid metabolism by PI3K/Akt/mTOR pathway, et al. (Lyu et al., 2018; Chen et al., 2019a).

The research team will continue to explore and verify the mechanism of differential metabolites in the production and progression of ovarian cancer ascites, as well as the mechanism of chemotherapy sensitivity and chemotherapy resistant in ovarian cancer ascites through *in vivo* and *in vitro* experiments, to find valuable biomarkers in the hope of guiding clinical diagnosis and treatment.

## Conclusion

In summary, this study had a comprehensive understanding of the metabolic changes of ovarian cancer tissues and ascites during chemotherapy and recurrence, and the potential differential metabolites can be used to reflect the prognosis of ovarian cancer patients and the extent of liver and kidney injury. The diagnostic and prognostic biomarkers of ovarian cancer screened by metabonomics provided reliable indicators for the prognosis, including postoperative ascites and ascites chemotherapeutic resistance, and can provide new ideas for the timely revision of treatment regimens.

## Data Availability

The original contributions presented in the study are included in the article/Supplementary Material, further inquiries can be directed to the corresponding author.
