# Supplementary figures and images for: Clinical Significance of Screening Differential Metabolites in Ovarian Cancer Tissue and Ascites by LC/MS
Source: Front Pharmacol. 2021 Nov 1;12:701487. doi: 10.3389/fphar.2021.701487 (PMC8593816; doi:10.3389/fphar.2021.701487)

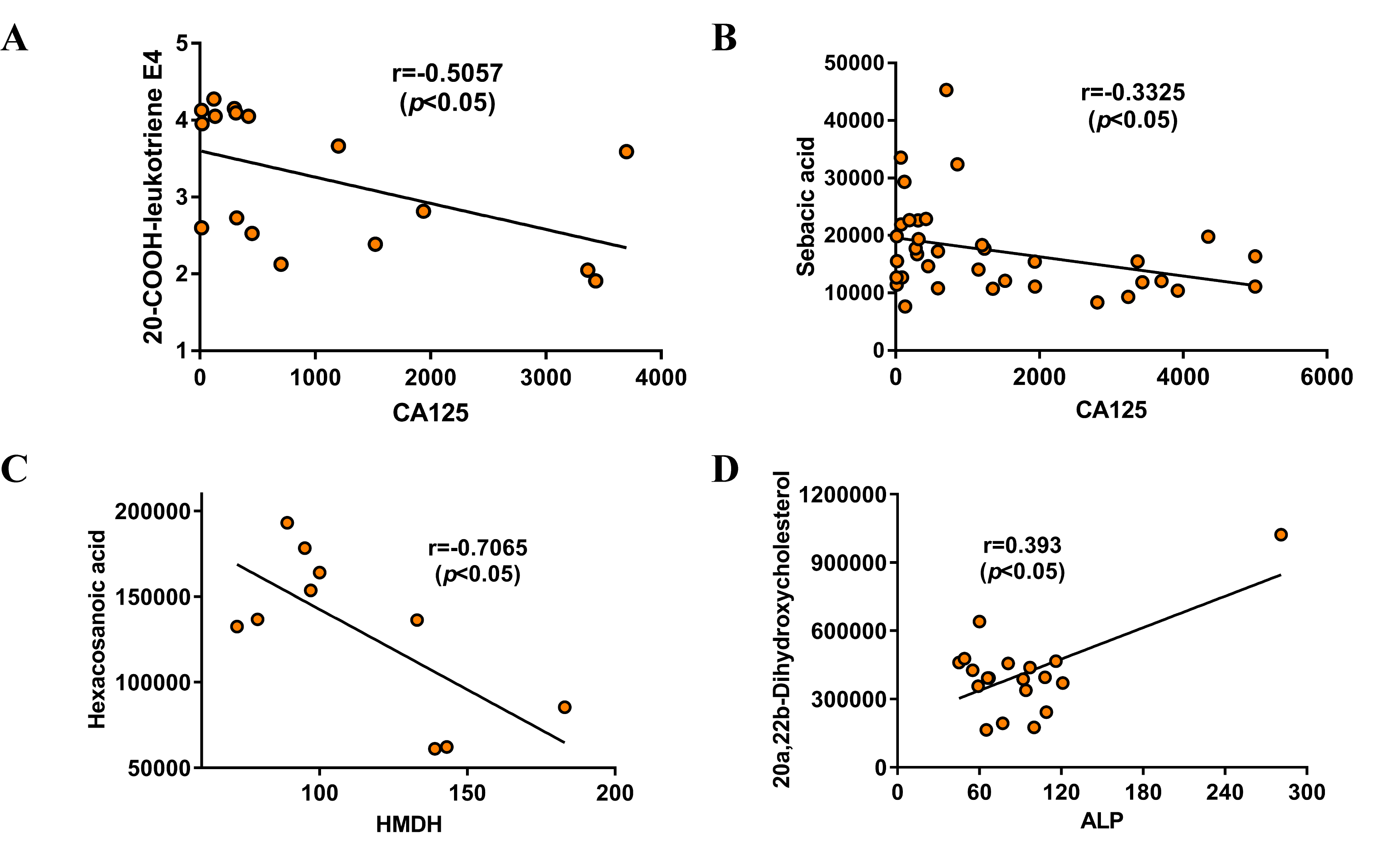

Supplement: Supplementary file 3 [file Image3.tif]

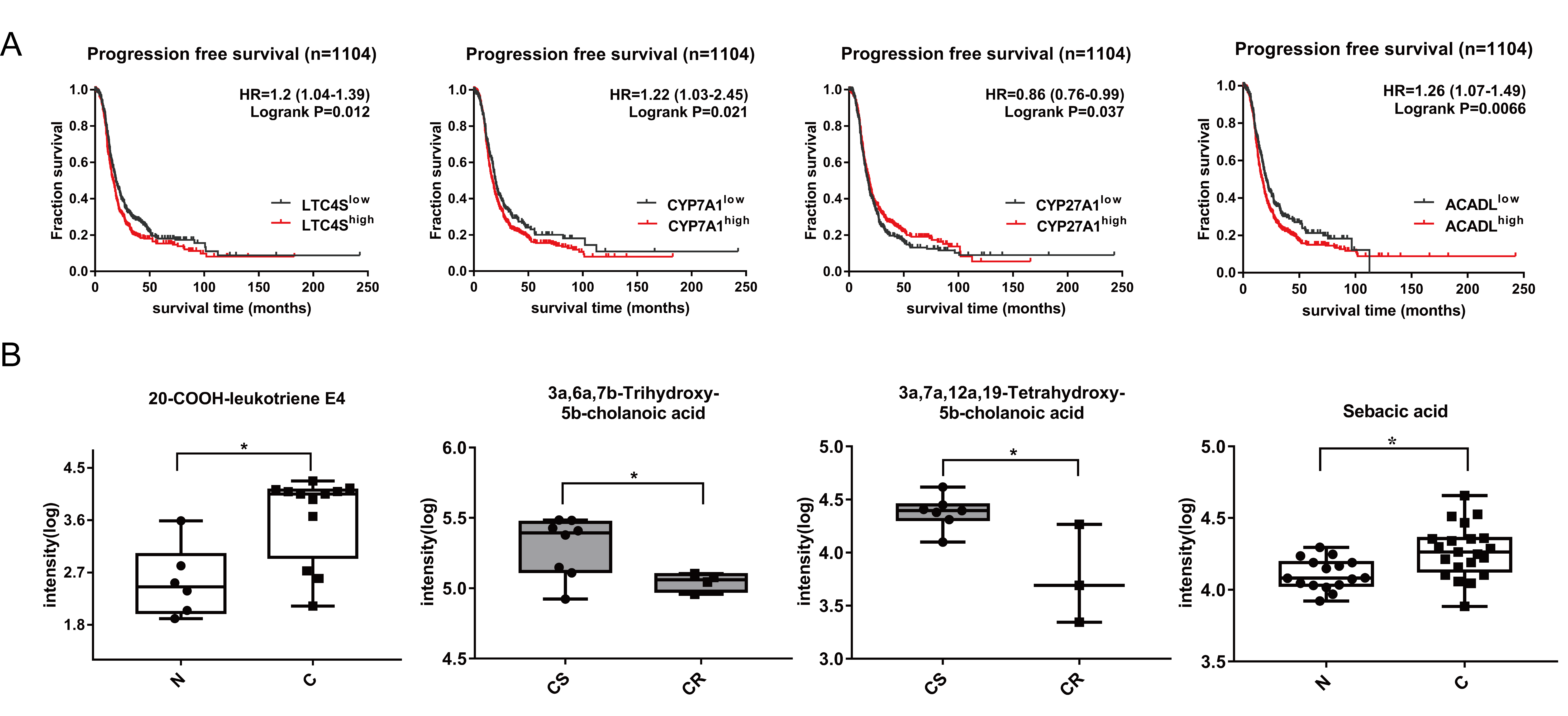

Supplement: Supplementary file 4 [file Image2.TIF]

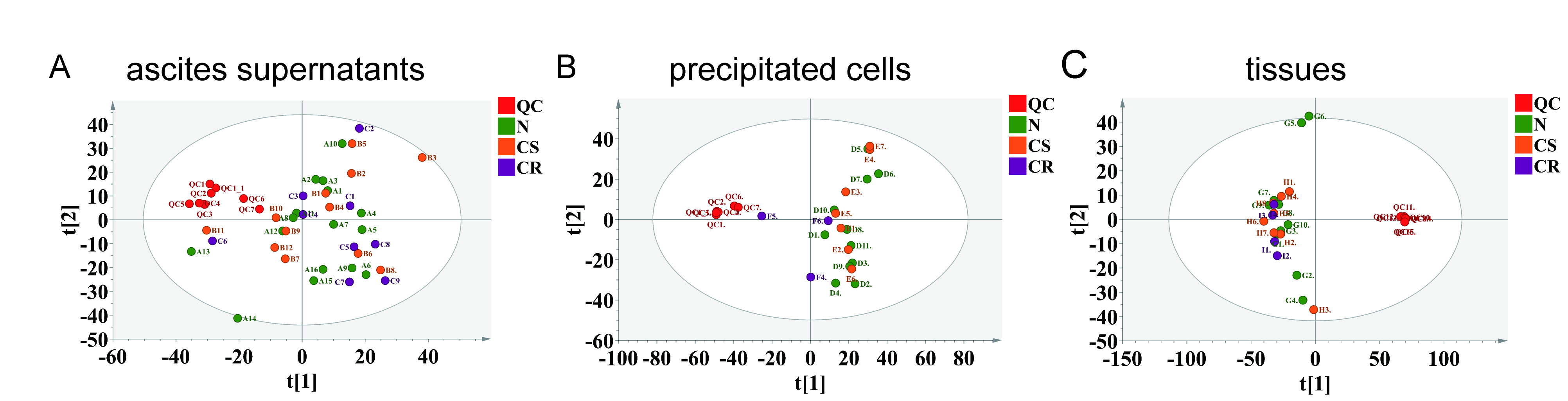

Supplement: Supplementary file 5 [file Image1.TIF]
